# Retraction: Gamification of Incentive Spirometry in Trauma Patients: Protocol for a Prospective, Observational Feasibility Study

**DOI:** 10.2196/109242

**Published:** 2026-08-19

**Authors:** JMIR Publications Editorial Office

**Affiliations:** 1 JMIR Publications Toronto, ON

The JMIR Publications Editorial Office is retracting the article “Gamification of Incentive Spirometry in Trauma Patients: Protocol for a Prospective, Observational Feasibility Study” [1] due to concerns regarding the involvement of a third party during the submission process.

Author Areen Al-Dhoon responded, stating that the identified individual did not contribute to the manuscript. Authors Aarti Sarwal, Carma Goldstein, Peter Spronk, R Shayn Martin, and Arthur Grimes expressed that they had no knowledge of these circumstances at the time of submission or revision. However, the Editorial Office has determined that there is sufficient evidence that the submission process was compromised to proceed with a retraction due to a loss of editorial confidence.

The authors disagreed with the decision to retract the article.
